# Subjective Cognitive Impairment, Depressive Symptoms, and Fatigue after a TIA or Transient Neurological Attack: A Prospective Study

**DOI:** 10.1155/2017/5181024

**Published:** 2017-11-19

**Authors:** Frank G. van Rooij, Nicole O. Plaizier, Sarah E. Vermeer, Bozena M. Góraj, Peter J. Koudstaal, Edo Richard, Frank-Erik de Leeuw, Roy P. C. Kessels, Ewoud J. van Dijk

**Affiliations:** ^1^Department of Neurology, Donders Institute for Brain, Cognition and Behaviour, Centre for Neuroscience, Radboud University Medical Center, P.O. Box 9101, 6500 HB Nijmegen, Netherlands; ^2^Department of Medical Psychology, Donders Institute for Brain, Cognition and Behaviour, Centre for Neuroscience, Radboud University Medical Center, P.O. Box 9101, 6500 HB Nijmegen, Netherlands; ^3^Department of Neurology, Rijnstate Hospital, P.O. Box 9555, 6800 TA Arnhem, Netherlands; ^4^Department of Radiology, Donders Institute for Brain, Cognition and Behaviour, Centre for Neuroscience, Radboud University Medical Center, P.O. Box 9101, 6500 HB Nijmegen, Netherlands; ^5^Department of Neurology, Erasmus Medical Center, P.O. Box 2040, 3000 CA Rotterdam, Netherlands; ^6^Donders Institute for Brain, Cognition and Behaviour, Centre for Cognition, Radboud University, P.O. Box 9101, 6500 HB Nijmegen, Netherlands

## Abstract

**Introduction:**

Subjective cognitive impairment (SCI), depressive symptoms, and fatigue are common after stroke and are associated with reduced quality of life. We prospectively investigated their prevalence and course after a transient ischemic attack (TIA) or nonfocal transient neurological attack (TNA) and the association with diffusion-weighted imaging (DWI) lesions.

**Methods:**

The Cognitive Failures Questionnaire, Hospital Anxiety and Depression Scale, and Subjective Fatigue subscale from the Checklist Individual Strength were used to assess subjective complaints shortly after TIA or TNA and six months later. With repeated measure analysis, the associations between DWI lesion presence or clinical diagnosis (TIA or TNA) and subjective complaints over time were determined.

**Results:**

We included 103 patients (28 DWI positive). At baseline, SCI and fatigue were less severe in DWI positive than in DWI negative patients, whereas at follow-up, there were no differences. SCI (*p* = 0.02) and fatigue (*p* = 0.01) increased in severity only in DWI positive patients. There were no differences between TIA and TNA.

**Conclusions:**

Subjective complaints are highly prevalent in TIA and TNA patients. The short-term prognosis is not different between DWI-positive and DWI negative patients, but SCI and fatigue increase in severity within six months after the event when an initial DWI lesion is present.

## 1. Introduction

Subjective cognitive impairment (SCI), depressive symptoms, and fatigue are highly prevalent after stroke and are related to stroke severity [1–4]. Although by definition the symptoms of a transient ischemic attack (TIA) subside completely within 24 hours [5], subjective cognitive complaints, depressive symptoms, and fatigue often persist in these patients as well [6–8].

Diagnosing TIA is notoriously difficult [9]. Patients often report attacks of atypical or nonfocal neurological symptoms. In the absence of an alternative diagnosis, these episodes are referred to as transient neurological attack (TNA) [10]. In one-third of TIA patients, diffusion-weighted imaging (DWI) shows signs of acute ischemia beyond the point of symptom resolution, ascertaining a cerebrovascular etiology of the attack [11]. Acute DWI lesions are however also present in more than 20% of clinically diagnosed TNA patients [12].

Previous studies on subjective complaints after short-lasting attacks of neurological symptoms have focused solely on TIA, did not take DWI findings into account, and were cross-sectional in nature [6–8]. Since subjective complaints are associated with a reduced quality of life and are possible harbingers of forthcoming cognitive decline, it is important to understand their prevalence and determinants [13, 14].

We prospectively investigated the prevalence, severity, and course of SCI, depressive symptoms, anxiety, and fatigue in a cohort of TIA and TNA patients and determined the relation with event type and DWI results. We hypothesized that patients with acute DWI lesions would report an increase in severity of complaints in the months after the initial event. Since TIA and TNA have a comparable prevalence of DWI positivity, we expected to find no differences between these patient categories [12].

## 2. Methods

### 2.1. Study Design and Patients

This study was part of the prospective Cohort study ON Neuroimaging, Etiology, and Cognitive consequences of Transient neurological attacks (CONNECT), the details of which have been previously reported [15]. Consecutive stroke-free patients aged ≥ 45 years referred to a specialized outpatient TIA clinic within 7 days after an event of acute onset neurological symptoms lasting < 24 hours were included. Baseline measurements took place within seven days after the qualifying event, and follow-up was performed six months later. All baseline assessments were performed before the final diagnosis was discussed with the patient. The Medical Review Ethics Committee region Arnhem-Nijmegen approved the study, and written informed consent was obtained from all participants.

### 2.2. Classification of Qualifying Event

Based on a detailed description of the signs and symptoms of the event including a structured assessment of the presence or absence of eighteen specific predefined symptoms (Table 1), three specialized stroke neurologists adjudicated each event as TIA, TNA, or a specified other diagnosis, using previously determined definitions of TIA and TNA [5, 10]. Events with both focal and nonfocal symptoms were classified as TIA. Qualifying neurologists were blinded to results from MRI, and in case of disagreement, a consensus meeting was held.

### 2.3. Brain Imaging

Brain MRI was performed within seven days after the qualifying event on a 1.5 Tesla Magnetom Scanner (Siemens, Erlangen, Germany) and included DWI, FLAIR, T1-, T2-, and T2^∗^-weighted sequences. Two experienced raters individually evaluated the severity of white matter hyperintensities, the presence of (silent) territorial infarcts, lacunes, and microbleeds [16, 17]. DWI was visually assessed for signs of acute infarction. Raters were unaware of clinical information, and a consensus meeting was held in case of disagreement.

### 2.4. Subjective Cognitive Impairment

At baseline and follow-up, the presence and severity of SCI in the previous month was assessed with a 15-item semistructured interview based on the Cognitive Failures Questionnaire (CFQ) [18]. Items concerning remembering, word finding, planning, concentration, and slowness of thought were given a wider score range (0–3) than other items (0-1). SCI was considered present if ≥1 moderate problem (score ≥ 2) on an item with a score range of 0 to 3 or a score of 1 on a dichotomous item was reported [19]. Trained examiners, unaware of clinical diagnosis and DWI status, administered all semistructured interviews.

### 2.5. Other Measurements

At both baseline and follow-up, the Hospital Anxiety and Depression Scale (HADS) was administered to measure the severity of symptoms of depression and anxiety [20]. Relevant symptoms of depression or anxiety were defined as a value of >7 on the subscales [21]. Fatigue was assessed with the subscale Subjective Fatigue of the Checklist Individual Strength (CIS20R-fatigue) with a score > 35 considered indicative for severe fatigue [22]. Level of education was classified using seven categories (1 = less than primary school; 7 = academic degree) [23]. Vascular risk factors were assessed at baseline. Incident vascular events (stroke, TIA, and myocardial infarction) between baseline and follow-up were assessed with a standardized, structured questionnaire. All questionnaires were handed out at the baseline or follow-up visit. In case of limited time, these were filled in at home and returned within one week.

### 2.6. Statistical Analysis

Only patients who completed follow-up were included. Baseline characteristics were compared between patients with complete and incomplete follow-up, between TIA and TNA between patients, and patients with and without DWI lesion, using Student's *t*-test, *χ*^2^ test, or Mann–Whitney *U* test as appropriate.

Differences in prevalence of SCI, symptoms of depression or anxiety, and severe fatigue between groups (clinical diagnosis and DWI status) and time points were analyzed with McNemar's test. Subsequently, the effect of DWI lesion and clinical diagnosis on change in subjective outcomes over time was determined with repeated measures analyses of variance. Associations between SCI, fatigue, and depressive and anxiety symptoms were assessed with multivariate regression analysis.

Age, sex, and level of education were regarded as potential confounders and adjusted for in all repeated measures analyses. Alpha was set at 0.05 and statistical analyses were performed with IBM SPSS Statistics version 24.0 (IBM Corp., Armonk, NY).

## 3. Results

**Figure 1:**
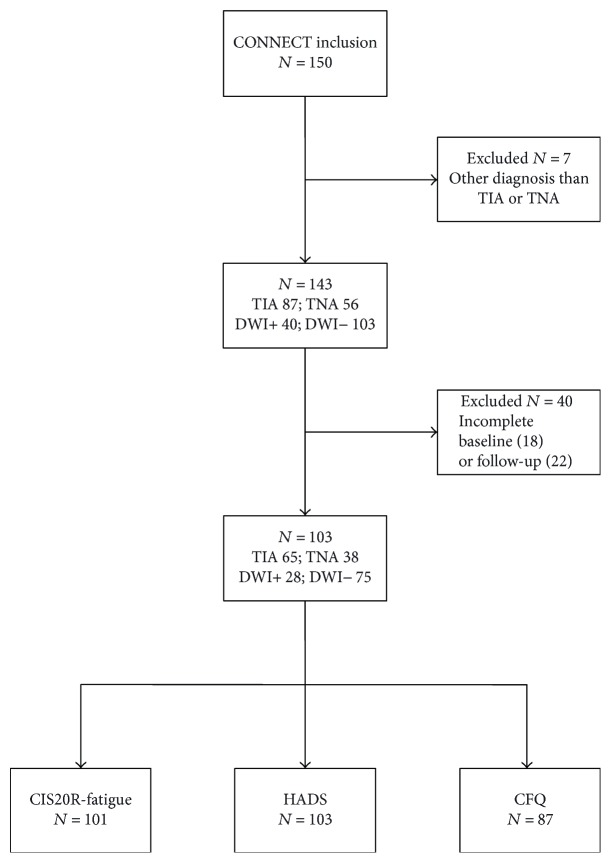
Study population. CFQ: Cognitive Failures Questionnaire; CIS20R-fatigue: Checklist Individual Strength, fatigue subscale; DWI: diffusion-weighted imaging; HADS: Hospital Anxiety and Depression Scale; TIA: transient ischemic attack; TNA: transient neurological attack.

CONNECT included 150 patients, 87 of whom were diagnosed as TIA, 56 as TNA, and 7 with a specific diagnosis. Patients in the last category were excluded from further analyses, as were those with incomplete baseline or follow-up assessments, leading to 103 included patients (Figure 1). Reasons for incomplete assessment were failure to return questionnaires (>95%) and refusal (<5%). Patients with incomplete HADS or CIS20R-fatigue were slightly younger (mean 61.8 [SD 10.7] years versus 65.8 [SD 9.1] years, *p* = 0.04) than those with complete evaluations. There were no differences concerning clinical diagnosis, DWI lesion presence, level of education, or presence of vascular risk factors between patients with complete and incomplete assessments. Baseline characteristics of participants with complete assessments are presented by DWI results in Table 2. DWI lesions were more often present in clinically defined TIA patients, although 13% of included TNA patients also had a DWI lesion. Most DWI lesions were small cortical or subcortical lesions. There were no incident strokes or myocardial infarctions between baseline and follow-up, and five patients had an incident TIA. Excluding patients with incident TIA from the analyses did not change the results.

### 3.1. Subjective Cognitive Impairment (*n* = 87)

**Figure 2:**
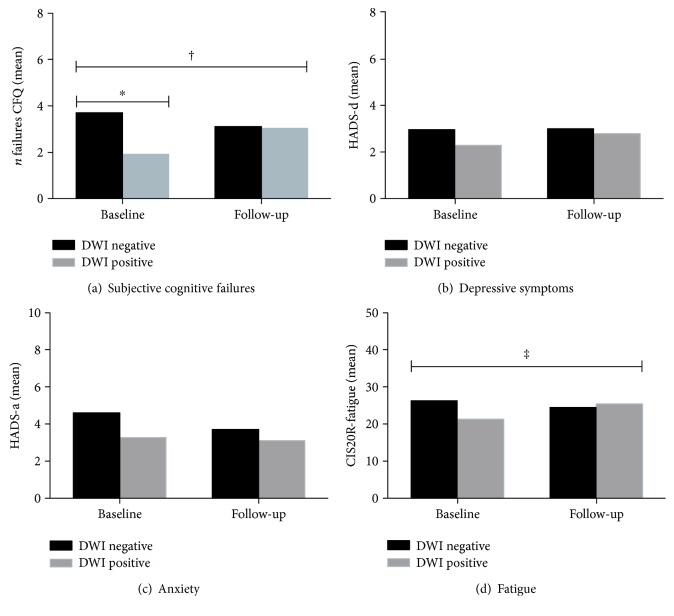
Subjective cognitive impairment, depressive symptoms, anxiety symptoms, and fatigue at baseline and follow-up in transient neurological attack patients with and without diffusion-weighted imaging lesions. CFQ: Cognitive Failures Questionnaire; CIS20R-fatigue: Checklist Individual Strength, fatigue subscale; DWI: diffusion-weighted imaging; HADS-a: Hospital Anxiety and Depression Scale, anxiety; HADS-d: Hospital Anxiety and Depression Scale, depression. ^∗^*p* = 0.02, analysis of covariance, adjusted for age, sex, and level of education. ^†^*p* = 0.02, ^‡^*p* = 0.01, repeated measures analysis for the difference in change over time between DWI negative and DWI positive patients, adjusted for age, sex, and level of education.

The overall prevalence of SCI in TIA and TNA patients was 82% at baseline and 77% at follow-up and was not significantly different between time points, DWI status, or clinical diagnosis. At baseline, the mean number of subjective cognitive failures was lower in patients with a DWI lesion than in those without (mean (SD) 1.83 (1.75) versus 3.77 (3.27), *p* = 0.01), while at follow-up, this increased to 3.00 (2.70) in the first group and decreased slightly to 3.14 (3.17) in the latter (*p* = 0.73) (Figure 2(a)). Repeated measures analysis (adjusted for age, sex, and level of education) showed that change over time in the number of subjective cognitive failures was significantly different between DWI positive and DWI negative patients (*p* = 0.01). There was no difference in SCI between TIA and TNA patients.

### 3.2. Depressive Symptoms and Anxiety (*n* = 103)

Relevant depressive symptoms (HADS-depression subscore > 7) were present in 8% of all patients at baseline and 9% at follow-up. The prevalence of relevant anxiety symptoms (HADS-anxiety subscore > 7) was 15% at baseline and 11% at follow-up. Depressive and anxiety symptoms did not differ between DWI negative and DWI positive patients or between TIA and TNA patient groups (Figures 2(b) and 2(c)).

### 3.3. Fatigue (*n* = 101)

Severe fatigue (CIS20R-fatigue score > 35) was present in 23% of patients at baseline and in 19% six months later. Prevalence of severe fatigue did not differ between DWI positive and DWI negative patients or between TIA and TNA patients.

Mean (SD) CIS20R-fatigue score was 25.0 (12.7) at baseline and 24.8 (11.5) at follow-up. Baseline scores were lower in DWI positive patients (21.0 (12.7) compared to 26.6 (12.4) for those without; *p* = 0.07) but equal at follow-up (24.9 (11.6) in the DWI positive group versus 24.7 (11.6) in the DWI negative group; *p* = 0.86). Patients with and without DWI lesions differed with respect to change in CIS20R-fatigue scores (*p* = 0.01) (Figure 2(d)). The clinical diagnosis was unrelated to severity or change over time of fatigue.

At both baseline and follow-up, SCI was associated with higher CIS20R-fatigue scores (*p* = 0.001), but not with symptoms of depression or anxiety.

## 4. Discussion

Subjective complaints, especially SCI and fatigue, are highly prevalent in TIA and TNA patients both directly before the event and after six months. The initial qualifying diagnosis was unrelated to the presence, severity, and course of subjective complaints. Patients with signs of acute ischemia on DWI reported less severe SCI and fatigue in the month before the TIA or TNA than those without such a lesion. In this group of patients, severity subsequently increased in the six months after the event to a level equal to that of DWI negative patients.

Some methodological issues need to be considered when interpreting these results. First, given the loss to follow-up, selection bias might have occurred. Patients with missing follow-up HADS and CIS20R-fatigue assessments were on average slightly younger than participants. Since especially fatigue is more often reported in older patients, this might have resulted in an overestimation of its prevalence [24]. Alternatively, patients with incomplete assessments might have dropped out because of complaints, resulting in an underestimation. Other demographic variables however did not differ between patients with and without complete assessments, and we adjusted for age in our analyses. Therefore, we feel that selection bias has not largely influenced our results. The relatively small patient numbers in our study however limit statistical power, and our results need to be replicated in a larger cohort. Secondly, we used questionnaires to obtain information on the presence of SCI, depression, anxiety, and fatigue. These screening instruments, although validated, indicate whether patients experience dysfunction when actively asked about it. This differs from spontaneously reported complaints and may explain the high frequency of SCI observed in our cohort. The CFQ handles a strict cutoff for SCI, making it a sensitive but perhaps not very specific screening instrument. Thirdly, our study did not include a control group, limiting the interpretation of an added effect of TIA or TNA on subjective complaints.

The prevalence of SCI in our study is comparable to that in both stroke patients and those with evidence of small vessel disease on neuroimaging, using the same screening instrument [2, 19, 25]. Patients in those studies were on average older, had more often suffered stroke instead of TIA, and were tested several years after the initial event. Also, vascular lesions on neuroimaging were relatively sparse in our cohort, as compared to these studies [19, 25]. The high prevalence of SCI in TIA and TNA patients is therefore remarkable. Severe fatigue was less prevalent in our patients than in other stroke cohorts, as were depressive symptoms [4, 26, 27].

Interestingly, DWI positive patients reported less subjective cognitive failures and fatigue in the month before the event than those without a lesion. The DWI positive group by definition consisted of patients with a recent cerebrovascular event, independent of the clinical diagnosis. The DWI negative group possibly included some patients whose transient complaints have noncerebrovascular causes such as somatization, depression, or anxiety. Although this may explain the higher prevalence of premorbid SCI and fatigue, the lack of observed higher frequencies of depressive or anxiety symptoms does not support this hypothesis. Furthermore, we found no changes in subjective complaints in the DWI negative group, although one could expect these to increase after a disturbing event such as a TIA or TNA.

Within six months after TIA or TNA, severity of SCI and fatigue increased significantly in DWI positive patients. This can be explained in several ways. First, DWI hyperintensities are associated with permanent brain damage, which in turn is associated with cognitive decline, SCI, mood disorders, and fatigue in stroke patients [1, 3, 4, 28, 29]. Although the exact etiology remains to be established, a relationship between minor cognitive decline and recent lacunar infarct has been suggested [30]. Since the DWI lesions in our cohort were predominantly small cortical and subcortical lesions, a similar relationship may exist between these lesions and increasing subjective complaints over time. Second, the knowledge of having a DWI lesion could have influenced the perception of cognitive performance and have led to more subjective cognitive complaints. Third, secondary preventive medications including statins will more often have been started in the DWI positive patient group. Statins have been associated with cognitive complaints and fatigue. However, there are no consistent negative effects of statins on these outcome measures [31]. Finally, the increase in severity of SCI and fatigue observed in the DWI positive patient group could be merely a correction of an unexplained baseline difference, that is, regression towards the mean. However, no changes in prevalence or severity of these complaints were found in the DWI negative group, countering this explanation.

Fatigue was associated with subjective cognitive dysfunction. Possibly, both fatigue and SCI, as measured in our study, are expressions of the same underlying sense of unwell-being. We found no association between depressive symptoms and subjective cognitive dysfunction, suggesting that the increased severity of SCI observed in DWI positive patients was not influenced by mood changes.

## 5. Conclusions

Subjective complaints are highly prevalent in TIA and TNA patients. Larger sampled studies with longer follow-up need to determine whether subjective complaints last beyond six months after TIA or TNA and assess the course over time with respect to DWI lesion presence and the association with cognitive performance. Furthermore, the etiology of subjective cognitive impairment and fatigue after short-lasting cerebral ischemia and the association with radiological markers of cerebrovascular damage such as lacunes and cerebral atrophy should be subject to further research. Our results nevertheless add to the growing notion that TIA and TNA are more than just transient attacks but are associated with ongoing deficits and problems. This can be used to inform patients on the potential long-term prognosis of their TIA or TNA.

## Figures and Tables

**Table 1:** Predefined focal and nonfocal neurological symptoms.

Focal	Nonfocal
Hemiparesis	Decreased consciousness or unconsciousness
Hemihypesthesia	Confusion
Dysphasia	Amnesia
Dysarthria	Unsteadiness
Hemianopia	Nonrotatory dizziness
Transient monocular blindness	Positive visual phenomena
Hemiataxia	Paresthesias
Diplopia	Bilateral weakness of arms or legs
Vertigo	Unwell feelings^∗^

Symptoms should have sudden onset, rapid clearance, and duration of <24 hours. ^∗^Referable to the nervous system if the referring physician considered TIA but patients were unable to specify further.

**Table 2:** Baseline patient characteristics stratified by DWI result (*n* = 103).

	DWI+ (*n* = 28)	DWI− (*n* = 75)	*p* ^∗^
Women	8 (29)	29 (39)	0.34
Age, mean (SD)	66.7 (8.5)	65.6 (9.3)	0.60
Level of education, median (IQR)	5 (3)	5 (2)	0.10
TIA	23 (82)	42 (56)	0.01
Hypertension	25 (89)	60 (80)	0.27
Dyslipidemia	18 (64)	53 (71)	0.53
Diabetes mellitus	2 (7)	7 (9)	0.73
Atrial fibrillation	3 (11)	10 (13)	0.72
Smoking	13 (46)	17 (23)	0.02
Diffusion-weighted imaging lesions, total *n*	45	N/A	N/A
Lesion type			
Small cortical	26 (58)		
Small subcortical	14 (31)		
Territorial	5 (11)		
Fazekas score, median (IQR)	1 (1)	1 (1)	0.30
Lacunes	5 (18)	12 (16)	0.82
Territorial infarcts	2 (7)	8 (11)	0.59
Microbleeds (available for 72 patients)	1 (6)	6 (11)	0.49

Values are *n* (%) unless stated otherwise. ^∗^For difference using Student's *t*-test, *χ*^2^ test, or Mann–Whitney *U* test as appropriate. DWI: diffusion-weighted imaging; IQR: interquartile range; TIA: transient ischemic attack; N/A: not applicable.
